# Role of Early Surgery in Spontaneous Enterocutaneous Tubercular Fistulae

**DOI:** 10.4103/1319-3767.70623

**Published:** 2010-10

**Authors:** Mrinal Pahwa, Mohit Girotra

**Affiliations:** Maulana Azad Medical College and Associated L.N.J.P Hospital, New Delhi, India; 1 The Johns Hopkins University/Sinai Hospital Program in Internal Medicine, Baltimore, Maryland, USA. E-mail: girotra.mohit@gmail.com

Sir,

In support to the article by Singh *et al*. titled, “Spontaneous tubercular enterocutaneous fistula developing in the scar of a surgery done 14 years earlier,”[1] we share our experience of spontaneous tubercular fistulae. Enterocutaneous fistulae are mostly iatrogenic or malignancy-induced or due to inflammatory disorders like Crohn’s disease. Spontaneous enterocutaneous fistula tubercular in origin is actually a very rare entity.

**Figure 1:**
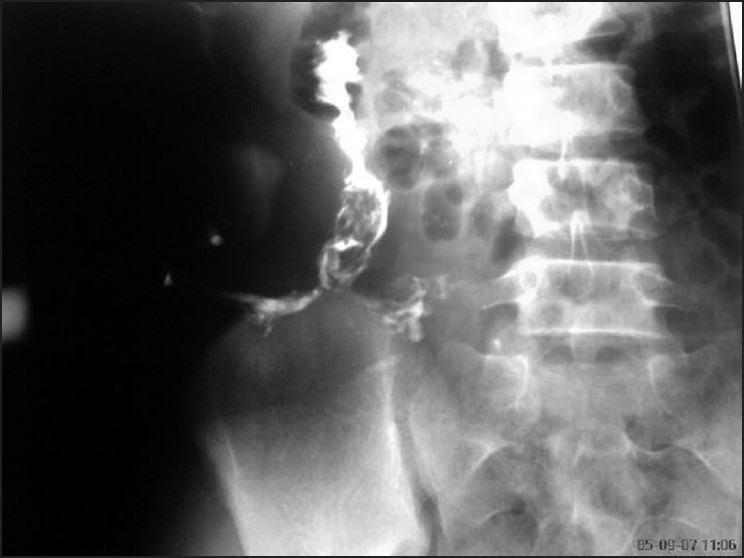
Fistulogram showing the enterocutaneous fistula

We recently encountered two patients, both females; one 36-year old presenting with complaints of altered bowel habits coupled with low grade fever and post-prandial fullness, and the other 15-year old with foul smelling discharge from an external opening in right loin. The physical exam, hematological, and biochemical parameters were essentially normal in both, except for high ESR. Mantaux test was positive in both, but chest and abdominal X-rays were normal. Diagnosis was clinched in both patients with abdominal ultrasound and barium meal follow through studies, all findings suggestive of tuberculosis. Fistulogram confirmed the communication of fistula with the bowel loops [Figure 1].

The patients were initiated on anti-tubercular therapy, beginning with intensive phase of four drugs for 2 months, followed by two drugs for 7 months. However, in both patients, the fistula did not heal even after completion of 9 months of treatment, hence warranting surgery. Exploratory laparotomy demonstrated findings of distal obstruction in the form of ileocaecal mass with fistula tract communicating with it. Hemicolectomy with ileotransverse anastomosis was performed and patients recovered uneventfully. The biopsy of the excised specimen from both patients revealed hyperplastic type of ileocaecal tuberculosis.

Our experience with these patients of spontaneous enterocutaneous fistulae due to tuberculosis makes a deviation from the accepted strategy of complete anti-tubercular treatment alone as the mainstay and consolidates our belief in advantage of early surgery in such patients. Fistulogram should be indispensible in all such cases to demonstrate the communication of fistula with the bowel. Once the diagnosis is confirmed and distal obstruction is ruled out, these patients should be initiated on conservative management with anti-tubercular therapy. However, from our experience, they often warrant surgery in due course of time as fistulae do not heal with conservative management alone and also some form of distal obstruction is often expected. Surgery should be in the form of removal of the diseased segment of the gut and restoration of intestinal continuity.
